# Co-production of single cell oil and gluconic acid using oleaginous *Cryptococcus podzolicus* DSM 27192

**DOI:** 10.1186/s13068-019-1469-9

**Published:** 2019-05-21

**Authors:** Xiujuan Qian, Olga Gorte, Lin Chen, Wenming Zhang, Weiliang Dong, Jiangfeng Ma, Min Jiang, Fengxue Xin, Katrin Ochsenreither

**Affiliations:** 1 State Key Laboratory of Materials-Oriented Chemical Engineering, College of Biotechnology and Pharmaceutical Engineering, Nanjing Tech University, Puzhu South Road 30#, Nanjing, 211816 People’s Republic of China; 2 Jiangsu National Synergetic Innovation Center for Advanced Materials (SICAM), Nanjing Tech University, Nanjing, 211816 People’s Republic of China; 3 Institute of Process Engineering in Life Sciences, Section II: Technical Biology, Karlsruhe Institute of Technology, Fritz-Haber-Weg 4, 76131 Karlsruhe, Germany

**Keywords:** Co-production, Single cell oil (SCO), Gluconic acid (GA), Regulation, Carbon flow

## Abstract

**Background:**

The co-production of single cell oil (SCO) with value-added products could improve the economic viability of industrial SCO production. The newly isolated oleaginous yeast *Cryptococcus podzolicus* DSM 27192 was able to co-produce SCO intracellularly and gluconic acid (GA) extracellularly. In this study, the metabolic regulation of carbon distribution between SCO and GA through process optimization was comprehensively investigated.

**Results:**

The carbon flow distribution between SCO and GA was significantly influenced by the cultivation conditions, such as nitrogen sources, glucose concentration and dissolved oxygen concentration. It was found that organic nitrogen sources were beneficial for SCO accumulation, while GA production was decreased. Dissolved oxygen concentration (DOC) was found to enhance SCO accumulation, while high glucose concentration was more favorable for GA accumulation. Hence, a two-stage DOC or glucose concentration-controlled strategy was designed to improve cell growth and direct carbon distribution between SCO and GA. Moreover, *C. podzolicus* DSM 27192 could degrade its stored lipids to synthesize GA in the late stationary phase, although considerable amounts of glucose remained unconsumed in the culture medium, indicating the importance of fermentation time control in co-production systems. All these observations provide opportunity to favor either the production of SCO or GA or rather their simultaneous production.

**Conclusions:**

Co-production of SCO and GA by *C. podzolicus* DSM 27192 can improve the economical value for microbial lipid-derived biodiesel production. Moreover, the results of the proposed co-production strategy might give guidance for other co-production systems.

## Background

Driven by the foreseeable depletion of crude oil and the highly controversial “food-or-fuel” discussion regarding plant-based biodiesel production, single cell oil (SCO) has been considered as an intriguing alternative for biodiesel production and oleochemical industries [1]. To unlock the economical competitiveness of SCO hindered by its relatively high production cost and low yield [2, 3], many efforts have been made on the utilization of low-cost feedstocks, optimization of fermentation conditions and genetic modification of lipid synthesis pathways in microbes [4–6]. However, the improvement is still limited, as the lipid yield and productivity are still too low [7–10]. Currently, the practical yield of SCO from glucose is only ~ 0.22 g lipid/g glucose, since high amounts of carbon are used for the production of biomass and other metabolites [11].

The production of some metabolites is inevitable for the maintenance of metabolism; alternatively, an effective co-production system may be more beneficial than the repression or deletion of genes related to by-products synthesis, as it may prevent metabolic imbalances [12]. In addition, the co-production of the main metabolic products, e.g., SCO with value-added chemicals, may make full use of nutrients in the cultivation medium, improving the techno-economics of microbial lipid technology [13]. Successful precedents have been established for the co-production of PHA with proteins, alcohols and biosurfactants [14]; β-galactosidase with ethanol [15]; erythritol with lipase [16]; lactic acid with chitin [17], etc. Currently, a number of co-production systems of SCO with other chemicals have also been reported, most of which have focused on the co-production with bioethanol [18], proteins [19] and some liposoluble products, such as carotenoid, fucoxanthin and pigments [20–22]. However, only little attention has been paid to the co-production of SCO with other bulk chemicals.

In our previous study, the oleaginous yeast *Cryptococcus podzolicus* DSM 27192 was isolated and identified. Preliminary fermentation results showed that it can accumulate high amounts of gluconic acid (GA) as a by-product when growing on glucose [23]. GA (C_6_H_12_O_7_) is an oxidation product of glucose, which has been widely used in the food, medicine and cement industries for over 50 years [24, 25]. Especially, the favorable effects of GA on human and animal health have boosted its use as a prebiotic in food production recently [26]. Due to its multiple applications in different aspects, the demand for GA has been steadily increasing globally. At present, the production of GA and its derivatives has been estimated to be approximately 60,000 ton/year, with production costs ranging from 1.20 to 8.50 US$/kg, which restricts its application in many cases [27]. Hence, developing an effective and economically viable system for GA production is urgent.

In this study, the co-production of SCO and GA by using *C. podzolicus* DSM 27192 was investigated. The influence of nitrogen source, dissolved oxygen, glucose concentration as well as fermentation time on the production of SCO and GA was comprehensively analyzed. Furthermore, biochemical and technological considerations concerning the microbial behavior were assessed and critically discussed, which will provide more guidance for further lipid co-production strategies.

## Results and discussion

According to literature, the production of GA and SCO is mainly influenced by three factors: (1) an excess of carbon; (2) nutrient limitation, e.g., nitrogen limitation; and (3) continuous aeration to ensure sufficient supply with oxygen. Therefore, these parameters were investigated accordingly [11, 24].

### Regulation of carbon flow between SCO and GA using different nitrogen sources

It is known that cell growth and distribution of metabolites are strongly affected by nitrogen sources during microbial fermentation [28]. In a previous shake flask study, SCO accumulation in *C. podzolicus* DSM 27192 was found to be similar in both YM and mineral salt medium. Furthermore, 1.8 g/L of GA was produced in mineral salt medium, while no GA was detected in YM medium (data not shown). A major difference between these two media was the nitrogen source. To further explore the effect of nitrogen sources on carbon flow distribution, different nitrogen sources including (NH_4_)_2_SO_4_, YM-based nitrogen (peptone, yeast extract and malt extract) and yeast extract were investigated in bioreactor experiments to ensure optimal aeration and pH control. To be able to compare the influence of different nitrogen sources, the C/N ratio was kept similar in all experiments as described by Li et al. [29] and Bellou et al. [30].

**Fig. 1:**
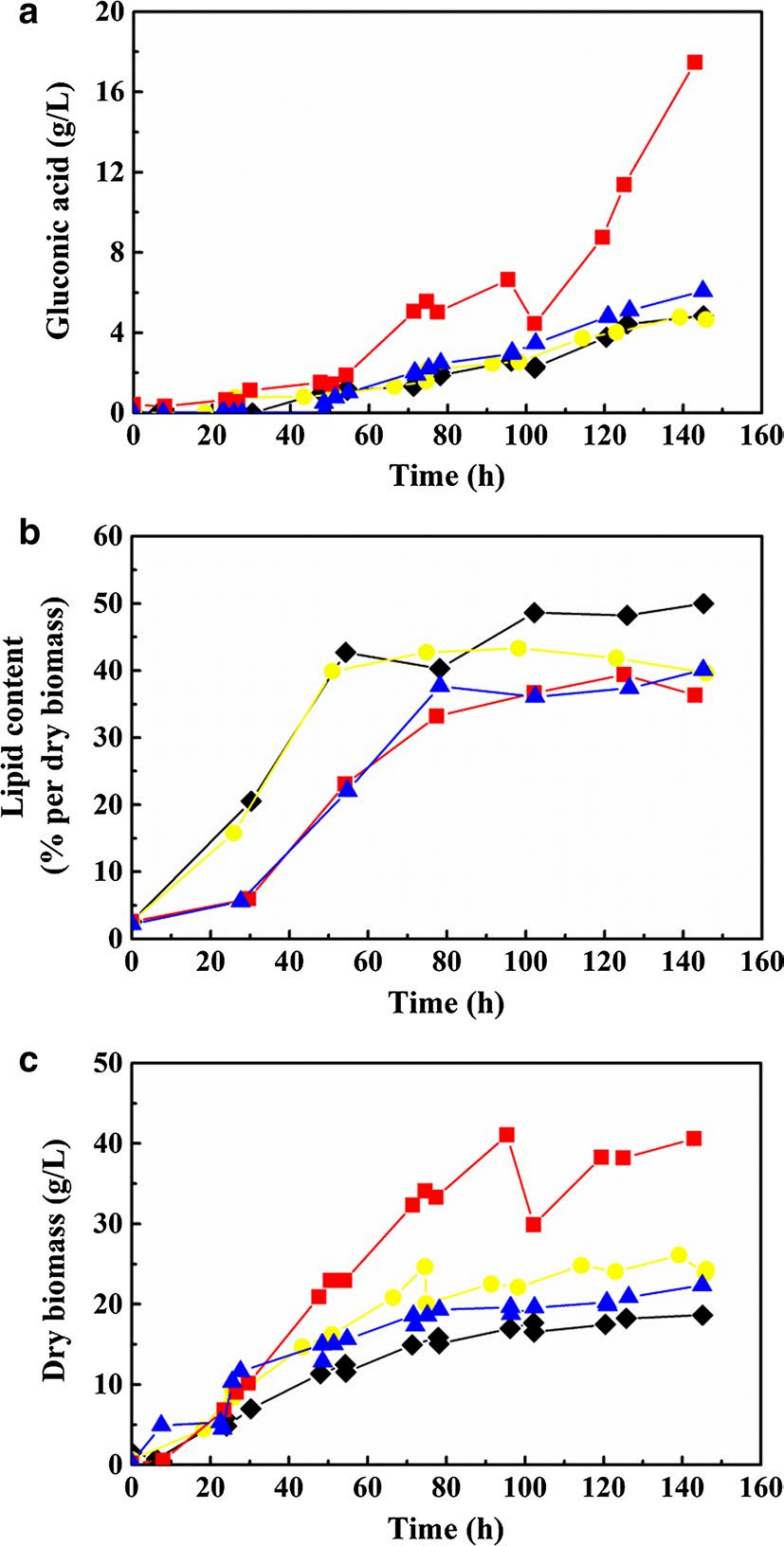
Influence of different nitrogen sources on the production of SCO and GA. **a** GA production; **b** SCO accumulation; **c** cell growth. Red diamonds represent fermented *C. podzolicus* DSM 27192 in mineral salt medium with (NH_4_)_2_SO_4_ as nitrogen source. Yellow dots and black rhombi represent *C. podzolicus* DSM 27192 grown in mineral salt medium with yeast extract and YM nitrogen as nitrogen source, respectively. The blue triangles show the properties of *C. podzolicus* DSM 27192 grown in YM medium. Experiments were done in a 2.5 L reactor, with 1.2 L of cultivation volume, 20 °C, 600 rpm agitation, and 1 vvm gas aeration. The cultures were held for ~ 150 h

As shown in Fig. 1a, when (NH_4_)_2_SO_4_ was used as the nitrogen source, 17.5 g/L of GA was produced by *C. podzolicus* DSM 27192, while considerably lower GA concentrations of 4.8 g/L, 4.6 g/L and 6.0 g/L were obtained in YM-based nitrogen, yeast extract medium and YM medium, respectively, suggesting a possible inhibition of organic nitrogen sources on GA production in contrast to inorganic ones. However, yeast extract is a complex mixture of nitrogenous compounds, carbon, sulfur, trace nutrients, vitamin B complex and other important growth factors. Thus, it is difficult to conclude which element(s) resulted in this phenomenon. In a previous study, some metals such as Cu^2+^ and Fe^2+^ in yeast extract have been reported to repress key enzyme activities, such as glucose oxidase (GO) in the GA production pathway [31–33]. However, the results are inconsistent with other studies, in which organic nitrogen sources, such as peptone and yeast extract, were found to have a stimulating effect on GO activity [34].

In terms of SCO production, organic nitrogen sources, such as yeast extract, were found to be favorable to improve the lipid content, since 49.9% and 39.6% were obtained in YM-based nitrogen and yeast extract medium, respectively. 40.1% was obtained by using YM medium, while a slightly lower lipid content of 36.3% was produced in (NH_4_)_2_SO_4_ medium (Fig. 1b). However, when (NH_4_)_2_SO_4_ was used as the nitrogen source, the highest biomass production (40.6 g/L) was detected (Fig. 1c), while YM medium, YM-based nitrogen and yeast extract medium resulted in 22.4 g/L, 18.6 g/L, and 24.3 g/L of cell mass, respectively. These results imply that inorganic and organic nitrogen play different roles in biomass production and lipid accumulation in the fermentation process, and that inorganic nitrogen sources are more beneficial for biomass accumulation, but less suitable for SCO production, while organic nitrogen sources improve lipid accumulation, but are less suitable for cell growth. These findings are consistent with the results reported by Bellou et al. [30]. Specifically, by measuring the enzyme activity of malic enzyme (ME) during *Yarrowia lipolytica* fermentation with different nitrogen sources, Bellou et al. found that ME may not be implicated in lipid biosynthesis in this yeast, and NADPH may be provided by the pentose phosphate pathway (PPP). The presence of organic nitrogen in low concentrations during lipogenesis was also required for NADPH supply of the lipogenic machinery in *Y. lipolytica*.

### Effect of glucose feeding on cell growth and carbon distribution

**Fig. 2:**
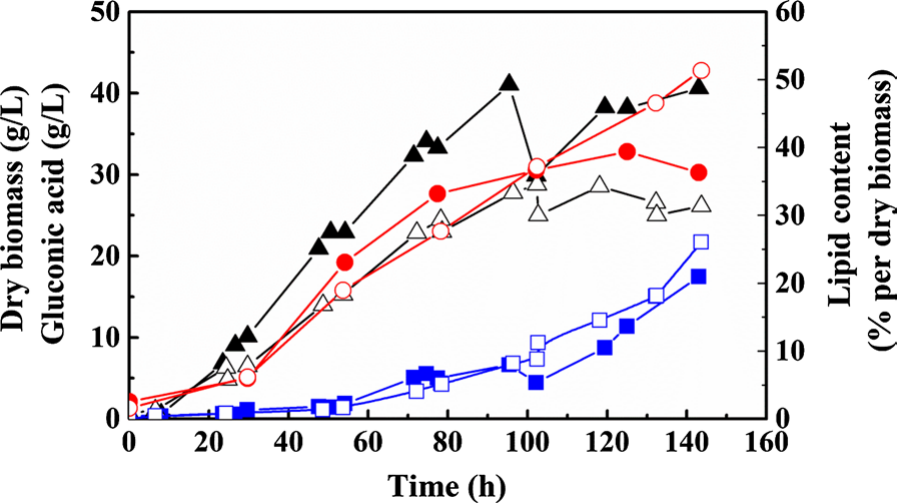
Influence of high glucose feeding on cell growth and the co-production of SCO and GA. The solid symbols represent the control group of mineral salt medium fermentation, in which 50 g/L of glucose was used as carbon source at the beginning, then up to 90 g/L of glucose was fed every day. The hollow symbols represent the group of high glucose fermentation, in which 90 g/L of glucose was used for cell growth at the beginning and then a fed-batch fermentation by feeding glucose up to 150 g/L every day was carried out. Triangles represent cell growth, diamonds represent gluconic acid production, and dots represent SCO accumulation. The fermentation was also carried out in a 2.5 L bioreactor with 1.2 L fermentation volume, and the cultivation condition was similar to that done previously

Excess carbon source is a prerequisite for SCO accumulation, and high glucose concentrations also induce the expression of GO. Accordingly, different glucose concentrations ranging from 9 to 15% were adopted to investigate the effect of glucose dosage on SCO accumulation and GA production. As seen in Fig. 2, 21.7 g/L of GA was produced when adding 15% glucose, with a yield of 0.16 g/g from glucose, corresponding to an increase of 24% compared with 9% glucose addition. However, an increased GA production due to higher glucose concentration might also cause more hydrogen peroxide accumulation in the medium, resulting in a severe damage on cell growth [32]. As observed in our experiment, the biomass did decrease by 35.5% when the glucose concentration was increased from 9 to 15%. In addition, cells generally tend to grow better at lower initial glucose concentration, since a high carbon source concentration might result in higher oxygen requirements during microbial cell growth, leading to a subsequent lower dissolved oxygen concentration (DOC) in the medium, which would force cells to enter the stationary growth phase prematurely [35].

With regard to the production of lipids, a content of 51.3% was obtained when the glucose concentration was increased from 9 to 15%, leading to a 41.3% improvement (Fig. 2). However, an increase in lipid content does not necessarily mean an increased lipid production in total. Since SCO is accumulated intracellularly, lower biomass concentration does discount SCO yield. As observed in this study, only 13.4 g/L of SCO was produced when 15% glucose was added, i.e., a 9% decrease compared to SCO production with 9% glucose. In conclusion, a high glucose concentration is more favorable for lipid accumulation rather than SCO production.

As expected, fatty acid composition was not affected markedly by varying the glucose concentration. The major fatty acids of *C. podzolicus* DSM 27192 were still palmitic acid (C16:0, ~ 20%) and oleic acid (C18:1, ~ 62%) (Table 1). Exceptionally, linoleic acid (C18:2) content did increase by 14.3%, from 7.7 to 8.8% in total lipids. A similar phenomenon was also reported for *Y. lipolytica*. When *Y. lipolytica* was cultivated with high initial glucose concentration, the concentration of cellular oleic acid decreased, while linoleic acid slightly increased [36]. This could be caused by an increased desaturation of oleic acid by Δ^12^desaturase within the fatty acid elongation cycle [37]. Therefore, optimizing the initial glucose concentration might be an easy way to upgrade SCO value by enhancing the content of polyunsaturated fatty acids in *C. podzolicus* DSM 27192 and other oleaginous yeasts.

**Table 1:** Fatty acid composition profile in % of total fatty acids of *C. podzolicus* DSM 27192 growing in a defined media with different glucose concentrations

Fatty acid species	9% Glucose 600 rpm	15% Glucose 600 rpm	9% Glucose pO_2_ > 40%	15% Glucose pO_2_ > 40%
Tetradecanoic acid (C 14:0)	0.0	0.1	0.1	0.1
Palmitic acid (C 16:0)	20.0	20.2	21.1	21.1
Octadecanoic acid (C 18:0)	5.5	5.3	5.5	5.5
Oleic acid (C 18:1)	62.9	61.9	61.5	61.5
Linoleic acid (C 18:2)	7.7	8.8	8.1	8.1
Arachidic acid (C 20:0)	0.7	0.8	0.8	0.8
Linolenic acid (C 18:3)	2.2	1.9	1.8	1.8
Docosanoic acid (C 22:0)	1.1	1.0	1.0	1.0
Erucic acid (C 22:1)	0.0	0.0	0.0	0.0
Lignoceric acid (C 24:0)	0.0	0.0	0.0	0.0

### Effect of DOC on cell growth and SCO–GA co-production

Dissolved oxygen concentration reflects the real-time state of cell growth and glucose uptake under a given agitation and aeration rate. In the early phase of *C. podzolicus* DSM 27192 cultivation, the DOC decreased rapidly to zero and remained zero throughout the exponential phase, suggesting that oxygen supply was a limiting factor for cell growth and lipid production, which was also observed when using other yeasts [35, 38]. Likewise, oxygen pressure was also proven to play a key role in GA production [39]. Therefore, further research regarding DOC influence on the production of SCO and GA was conducted.

**Fig. 3:**
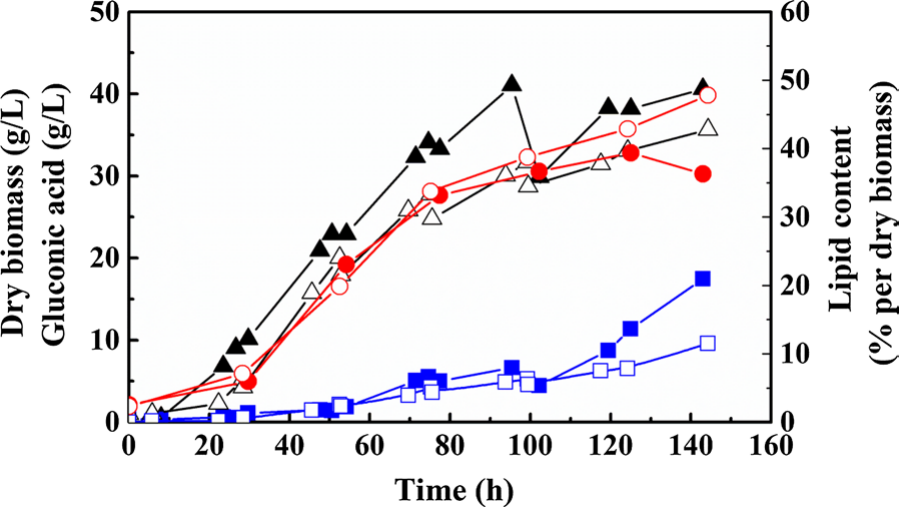
Influence of high DOC on cell growth and co-production of SCO and GA. The solid symbols represent the control group growing in mineral salt medium with a constant agitation speed at 600 rpm. The hollow symbols represent a high DOC fermentation, when DOC was kept over 40% by increasing the agitation mechanically. Triangles represent cell growth, diamonds represent gluconic acid production, and dots represent SCO accumulation. Experiments were done in a 2.5 L reactor, with 1.2 L of cultivation volume, 20 °C and 1 vvm gas aeration, and the cultures were held for ~ 150 h

The growth curve of *C. podzolicus* DSM 27192 cultivated with DOC above 40% as well as the production of SCO and GA is illustrated in Fig. 3. After 40 h of cultivation, the agitation speed was increased gradually from 600 rpm up to 930 rpm to keep DOC above 40%. The increased agitation resulted in a slight damage to cell growth, as 12% decrease in cell mass was found when compared with that under a constant agitation of 600 rpm. However, the negative influence of the increased agitation on GA production was more pronounced than on cell growth. GA production was maintained at a sluggish rate under high agitation condition, even after more glucose was supplemented. Only 9.6 g/L of GA was obtained after 145 h, with a GA yield of only 0.06 g/g glucose (Table 2), showing a 45% decrease compared with that under a constant agitation of 600 rpm. The reason could be that the activity of GO dropped sharply due to the increase in agitation speed as reported by Petruccioli et al. [40].

**Table 2:** Product titers and yields obtained at different fermentation parameters

Sugar addition (%)	Dissolved O_2_ (pO_2_)	Cell density (g/L)	Lipid content (% per CDW)	SCO production (g/L)	GA production (g/L)	Glucose consumption (g/L)	*X*_*L*/*g*_ (g SCO/g glucose)	*X*_*G*/*g*_ (g GA/g glucose)
9	600 rpm	40.6	36.3	14.7	17.5	156	0.09	0.11
15	600 rpm	26.2	51.3	13.4	21.7	140	0.10	0.16
9	pO_2_ > 40%	35.7	47.8	17.1	9.6	157	0.11	0.06
15	pO_2_ > 40%	21.8	53.8	11.7	15.4	122	0.10	0.13

*X*_*L*/*g*_ was estimated by the formula *X*_*L*/*g*_ = SCO production/glucose consumption

*X*_*G*/*g*_ was estimated by the formula *X*_*L*/*g*_ = GA production/glucose consumption

In contrast, the increased DOC had a positive effect on SCO accumulation, as a noticeable increase from 36.3 to 47.8% in lipid content was observed, corresponding to an increased lipid yield of 0.11 g/g glucose. This may be attributed to the upregulation of ATP-citrate lyase and malic enzyme at high DOC, which participate in lipid synthesis while the activity of NAD^+^-dependent isocitrate dehydrogenase was also found to decrease during the transition from low to high DOC in that study, usually a prerequisite for lipid accumulation [35]. Besides from *Y. lipolytica* and *Rhodotorula glutinis*, high DOC was beneficial for cell growth, but unfavorable for lipid accumulation in other studies [41, 42].

The changes of overall fatty acid composition under different DOC conditions were negligible (Table 1). For example, under constant agitation, the main fatty acids are palmitic acid (C16:0) 20.0%, stearic acid (C18:0) 5.5%, oleic acid (C18:1) 62.9%, and linoleic acid (C18:2) 7.7%. A nearly identical composition was obtained, when DOC was kept above 40% (21.1%, 5.5%, 61.5%, and 8.1% of C16:0, C18:0, C18:1 and C18:2, respectively). In contrast to *Lipomyces starkeyi* [8], an increased DOC did not decrease the saturation degree of fatty acids. Speculatively, the amount of oxygen required for the unsaturation is negligible when compared to cell propagation, energy metabolism, and total lipid biosynthesis in this yeast under current conditions.

### SCO and GA accumulation under high DOC and high glucose feeding at different fermentation stages

**Fig. 4:**
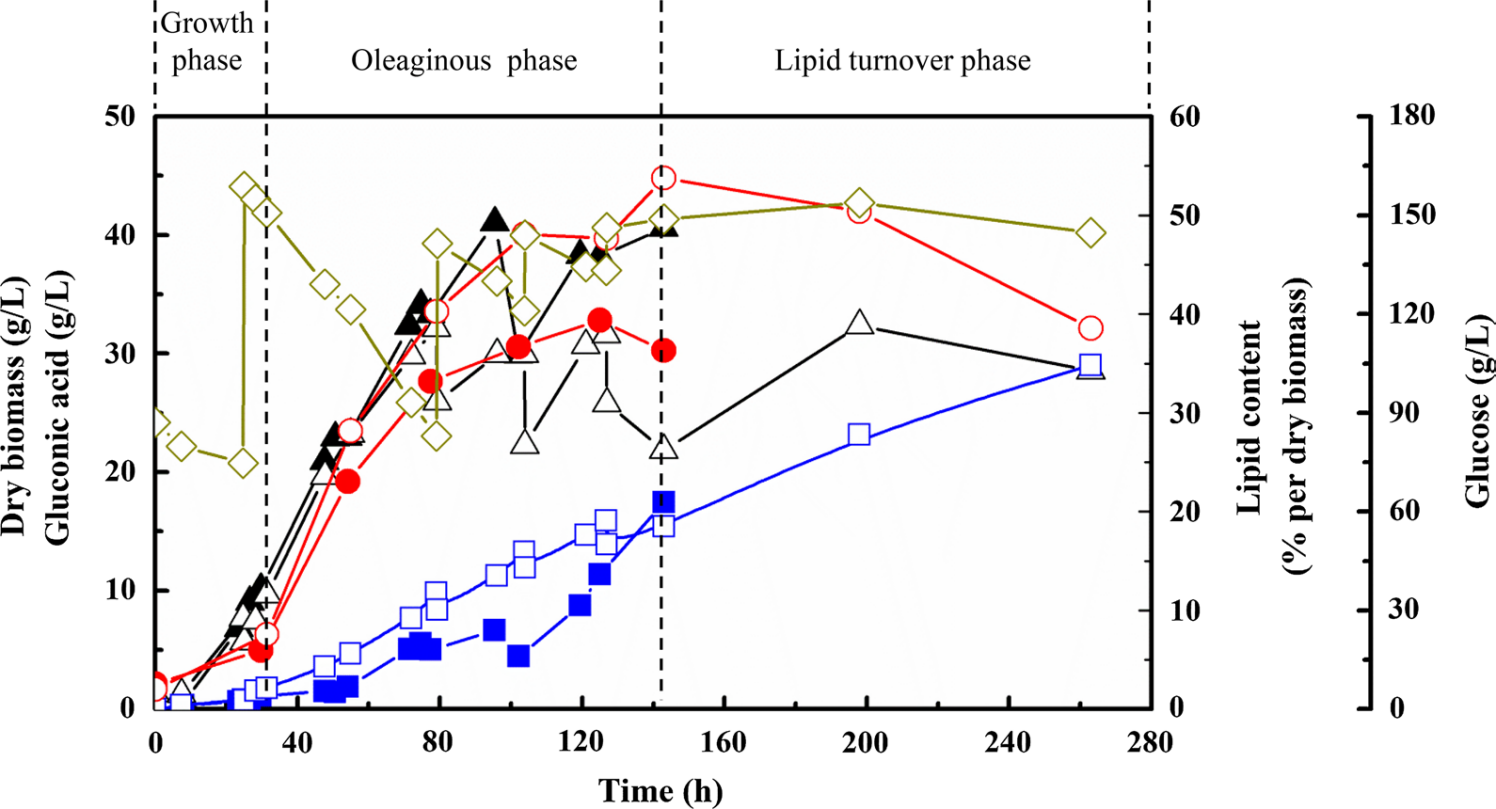
Kinetics of dry cell mass, gluconic acid production and lipid accumulation and turnover in *C. podzolicus* DSM 27192. The solid symbols represent the control group similar to the previous two parts. The hollow symbols represent a high glucose concentration and DOC fermentation, for which 90 g/L of glucose was used for cell growth at the beginning, then glucose was fed up to 150 g/L every day, and DOC was kept over 40% by increasing the agitation mechanically. Triangles represent cell growth, diamonds represent gluconic acid production, dots represent SCO accumulation, and rhombi represent the glucose concentration. Experiments were done in a 2.5 L reactor, with 1.2 L of cultivation volume and 20 °C and 1 vvm gas aeration. The cell growth phase ended with the depletion of nitrogen in the growth medium. The oleaginous phase started after nitrogen depletion and ended after 140 h fermentation. Then, the lipid turnover phase took place

Taken together, high DOC is favorable for SCO production, while high glucose concentration is favorable for GA production (Table 2). To explore the comprehensive effect of high DOC and high glucose addition on both cell growth and metabolites mainly SCO and GA production, experiments with high glucose feeding rate and high DOC above 40% were conducted. As expected, the growth of *C. podzolicus* DSM 27192 was severely inhibited by the increased glucose concentration and high agitation (Fig. 4). Only 21.8 g/L of cell mass was obtained after 142 h, resulting in the lowest lipid production of 11.7 g/L (Table 2). In addition, the neutralization caused by the high DOC and high glucose addition resulted in only 15.4 g/L of GA production.

It was found that cells grew fast during the growth phase, accumulating little SCO, with nearly no GA production. When the fermentation process entered the oleaginous phase after nitrogen depletion, glucose was channeled mainly to SCO accumulation, as relatively small amounts of GA were formed. At the end of the oleaginous phase, the cellular stored lipids were gradually degraded and presumably used for GA production, although glucose was still available in the medium (Fig. 4). By this strategy, it was possible to separate the production phases of both metabolites. Similar phenomena were also observed by using other oleaginous microorganisms. For example, when *Aspergillus niger* was cultivated on waste glycerol medium derived from the biodiesel industry, stored lipids were used to produce significant amounts of oxalic acid even under high glycerol concentration [43]. Also, stored lipids in *Y. lipolytica* were degraded and citric acid was produced despite high amounts of glycerol [44]. Generally, degradation of lipids for the production of other compounds occurs when carbon sources are completely exhausted [45, 46], or when the carbon source uptake rate cannot satisfy the anabolic activities inside the cell [44]. Especially during lipid accumulation, the uptake and metabolism of glucose are very slow when nitrogen is depleted [47]. Thereby, nitrogen limitation would induce lipid accumulation in the first growth stage, while the GA biosynthesis pathway was not completely activated. Afterward, stored lipids were degraded to synthesize GA due to the restriction in glucose transportation and metabolism. However, further analysis of the metabolic balance between SCO and GA using transcriptomics or proteomics is needed for future studies.

## Conclusion

Different factors influencing the carbon flow between SCO and GA production, namely nitrogen source, dissolved oxygen, glucose concentration and cultivation time, have been studied using *C. podzolicus* DSM 27192. The carbon distribution between SCO and GA was found susceptible to cultivation conditions. In detail, the GA production pathway was severely inhibited by organic nitrogen sources; high glucose addition greatly stimulated GA production, and both high glucose addition and high DOC were proven to promote SCO accumulation. However, either high glucose addition or high DOC had negative effects on cell mass growth, especially high glucose addition, which discounted the total SCO production. Hence, a two-stage DOC or glucose concentration control strategy was needed to improve cell growth and distribute carbon between SCO and GA. Moreover, the fermentation time is important for lipid production, since stored lipids will be degraded and used for the production of non-lipid metabolites in the late stationary phase. All these observations give the opportunity to favor either the production of SCO or GA or rather their simultaneous production. More importantly, this study might give more guidance for other co-production systems. Further studies should be focused on developing more efficient SCO co-production systems, improving the total product yield, amending the SCO production pathway, and enhancing specific high-value fatty acid production.

## Methods

### Microorganisms and medium

*Cryptococcus podzolicus* DSM 27192 used in this study was deposited at DSMZ culture collection in Braunschweig, Germany. The glycerol stocks were stored at − 80 °C in the in-house culture collection. Yeast colonies were maintained at 4 °C on Yeast Malt (YM) Agar plates (g/L): yeast extract 3.0, malt extract 3.0, peptone 5.0, glucose 10.0, agar 20.0, pH 6.2. For SCO production, a mineral salt medium was used as described by Ines Schulze et al. [23]. 50 g/L of glucose was used initially, from the 2nd day the glucose was replenished to a maximum concentration of 90 g/L after determining the actual concentration.

The nitrogen content was around 8–12% in yeast extract, 10–13% in peptone, and < 1% in malt extract used in this study. In the experiment of nitrogen influence study, to keep the C/N condition similar to the mineral salt medium, 12.1 g/L of YM nitrogen (3.3 g/L of yeast extract, 5.5 g/L of peptone, and 3.3 g/L of malt extract) and 10 g/L of yeast extract were used, respectively, to replace (NH_4_)_2_SO_4_ as the nitrogen source.

### Batch fermentation in bioreactor

A loopful of cells from YM agar plates was used to inoculate 20 mL of mineral salt medium (50 g/L of glucose as the carbon source) in a baffled shake flask and incubated in a gyratory shaker at 20 °C and 130 rpm for approximately 24 h. A second pre-culture was carried out using the same conditions. The second pre-culture was prepared from the first pre-culture in 200 mL culture medium in 2 L shake flasks with an initial OD_600_ of 1.0.

Fermentation was performed in a 2.5 L fermenter (Infors HT, Bottmingen, Switzerland; Minifors fermenter) with working volume of 1.2 L, an initial OD_600_ of 0.5–1.0 at 600 rpm, and 1 vvm aeration rate without control of dissolved oxygen level (pO_2_). The pO_2_ value was measured constantly by a probe. The control of pH to 6.0 was done automatically by the addition of 4 M H_3_PO_4_ and 4 M NaOH. In each fermenter, Contraspum A 4050 HAC (Zschimmerund Schwarz GmbH und Co KG, Lahnstein, Germany) was applied as antifoam agent. Each day, 20 mL of salt solution and 20 mL of trace element solution were fed into the medium.

All experiments were done in independent duplicates under the same condition.

### Determination of cell mass

The cell concentration in the culture medium was determined by the optical density at 600 nm (OD_600_) with the initial culture medium as blank. All samples were diluted to an optical density of 0.2–0.8 at 600 nm for measurement.

Cell dry biomass (CDW) was analyzed gravimetrically. 1 mL aliquot of the culture broth was transferred into a pre-dried and pre-weighed 1.5 mL reaction tube and centrifuged at 13,000 rpm for 5 min. The supernatant was collected and used for the determination of glucose, GA, and acetic acid. The cell pellet was washed with 1 mL saline (0.9% NaCl), dried at 60 °C for 24 h and weighed. For each sample, all parameters were measured in duplicate.

### Determination of glucose and GA

For glucose determination, 45 µL 4 M NH_3_Cl and 100 µL 1.2 M MgSO_4_ were added to 1 mL pure supernatant and subsequently centrifuged for 5 min at 20,000×*g* after 5 min of incubation. 500 µL supernatant was then transferred to 500 µL 0.1 M H_2_SO_4_, mixed, and incubated for 15 min. After the final centrifugation step of 15 min at 20,000×*g*, the supernatant was used for HPLC analysis. The analysis was performed with a standard HPLC device (Agilent 1100 Series, Agilent, Germany) with a Rezex ROA organic acid H^+^ (8%) column (300 by 7.8 mm, 8 m; Phenomenex) protected by a Rezex ROA organic acid H^+^ (8%) guard column (50 by 7.8 mm). Separation was performed under isocratic conditions at 50 °C (column temperature) for 45 min with 5 mM H_2_SO_4_ as the mobile phase at a constant flow rate of 0.4 mL/min. Detection of carbohydrate was achieved via an Agilent 1200 series refractive index detector at 50 °C.

For GA analysis, the untreated supernatant was collected and diluted to an appropriate concentration using 20 mM KH_2_PO_4_ (pH 2.5). The analysis was performed with a standard HPLC device (Agilent1100 Series, Agilent Technologies Deutschland GmbH, Böblingen, Germany) equipped with a 150 × 4.6 mm HPLC column Synergi™ 4 μm Fusion-RP 80 Å (Phenomenex, Aschaffenburg, Germany; 00 F-4424-E0) at 30 °C column temperature. 20 mM KH_2_PO_4_ (pH 2.5) (A) and 100% methanol (B) were used as eluents to drive the following temporal gradient: 0–0.5 min 100% eluent A, 0.5–10 min increase of eluent B from 0 to 10%,10–12 min decrease of eluent B from 10% back to 0% and 12–14 min again 100% eluent A. 10 μL sample was injected, a flow rate of 1 mL/min was adjusted, and peaks were detected via UV at 220 nm.

### Lipid extraction and determination of fatty acid methyl esters

20 mL aliquot of the culture broth was centrifuged (4700 rpm, 5 min), the pellet was resuspended in saline (0.9% NaCl). and centrifuged (4700 rpm, 5 min) again. The supernatant was discarded and the pellet was freeze dried (− 30 °C, 0.370 mbar). Sample preparation for the quantitative and qualitative gas chromatographic analysis was done in a one-step-procedure of direct esterification coupled with extraction. A portion (20 mg) of freeze-dried biomass was weighed into a 15 mL glass Falcon with Teflon cap. 1.5 mL of hexane and 0.5 mL of 2.0 mg/mL internal standard (methyl benzoate dissolved in hexane) were added as solvent for lipid extraction. In addition, 2 mL 15% H_2_SO_4_ in methanol was added for esterification. Then, the sample was heated up to 100 °C for 2 h with continuous shaking. After cooling on ice, 1 mL demineralized water was added, and the mixture was centrifuged for 5 min with 2500 rpm at 4 °C. Afterward, 1 μL of the upper phase was analyzed via chromatography (Agilent Technologies, 6890 N Network GC-System). The instrument was equipped with a DBWax column (Length: 30 m, diam: 0.25 mm, film: 0.25 μm; Agilent Technologies Deutschland GmbH, Böblingen, Germany; 122-7032) and a flame ionization detector and worked with a pressure of 1.083 bar and initial temperature of 40 °C. The column temperature was increased from 40 to 250 °C at a rate of 8 °C/min. The temperature was held at 250 °C for 10 min before cooling down to 40 °C. The total fatty acid content and the identification of fatty acids were obtained using the standard RM3 FAME Mix (Sigma Aldrich, Taufkirchen, Germany; 07256-1AMP) and Marine FAME Mix (Acid Methyl Ester Marine Oil FAME Mix) (Restek GmbH, Bad Homburg, Germany; 35066). Fatty acids which represented less than 1% of total fatty acids were combined to “trace fatty acids”.

## Data Availability

The information about accession numbers is given in the manuscript.

## Abbreviations

SCO: single cell oil
GA: gluconic acid
GO: glucose oxidase
DOC: dissolved oxygen concentration
